# Cyclin-Dependent Kinase 6 Phosphorylates NF-κB P65 at Serine 536 and Contributes to the Regulation of Inflammatory Gene Expression

**DOI:** 10.1371/journal.pone.0051847

**Published:** 2012-12-26

**Authors:** Holger Buss, Katja Handschick, Nadine Jurrmann, Pirita Pekkonen, Knut Beuerlein, Helmut Müller, Robin Wait, Jeremy Saklatvala, Päivi M. Ojala, M. Lienhard Schmitz, Michael Naumann, Michael Kracht

**Affiliations:** 1 Institute of Pharmacology, Medical School Hannover, Hannover, Germany; 2 Institute of Experimental Internal Medicine, Otto von Guericke University, Magdeburg, Germany; 3 Institute of Biotechnology and Research Programs Unit, Genome-Scale Biology, Biomedicum Helsinki, University of Helsinki, Helsinki, Finland; 4 Rudolf-Buchheim-Institute of Pharmacology, Justus-Liebig-University Giessen, Giessen, Germany; 5 The Kennedy Institute of Rheumatology Division, ARC Building, Charing Cross Campus, Imperial College London, London, United Kingdom; 6 Foundation for the Finnish Cancer Institute, Biomedicum Helsinki, University of Helsinki, Helsinki, Finland; 7 Institute of Biochemistry, Justus-Liebig-University Giessen, Member of the German Center for Lung Research, Giessen, Germany; National Cancer Institute (INCA), Brazil

## Abstract

Nuclear factor kappa-B (NF-κB) activates multiple genes with overlapping roles in cell proliferation, inflammation and cancer. Using an unbiased approach we identified human CDK6 as a novel kinase phosphorylating NF-κB p65 at serine 536. Purified and reconstituted CDK6/cyclin complexes phosphorylated p65 *in vitro* and in transfected cells. The physiological role of CDK6 for basal as well as cytokine-induced p65 phosphorylation or NF-κB activation was revealed upon RNAi-mediated suppression of CDK6. Inhibition of CDK6 catalytic activity by PD332991 suppressed activation of NF-κB and TNF-induced gene expression. In complex with a constitutively active viral cyclin CDK6 stimulated NF-κB p65-mediated transcription in a target gene specific manner and this effect was partially dependent on its ability to phosphorylate p65 at serine 536. Tumor formation in thymi and spleens of v-cyclin transgenic mice correlated with increased levels of p65 Ser536 phosphorylation, increased expression of CDK6 and upregulaton of the NF-κB target cyclin D3. These results suggest that aberrant CDK6 expression or activation that is frequently observed in human tumors can contribute through NF-κB to chronic inflammation and neoplasia.

## Introduction

The transcription factor nuclear factor kappa B (NF-κB) comprises homo-or heterodimeric combinations of five DNA-binding subunits which regulate the expression of a large number of genes in multiple physiological or pathophysiological conditions such as inflammation, immune reactions or cancer [1]. NF-κB activation is prevented by cytosolic retention by inhibitor of NF-κB (IκB) proteins. Phosphorylation-dependent proteolytic degradation of IκBs in response to inducers such as proinflammatory cytokines, in particular IL-1 or TNFα, is followed by nuclear translocation and DNA binding of NF-κB subunits. Thousands of potential DNA binding sites have been described across the genome [2]. Hence, NF-κB activity needs to be spatially, kinetically and quantitatively controlled to allow precise expression of its target genes [1]. Within the nuclear compartment, there is an additional layer of regulation of NF-κB activity provided by numerous posttranslational modifications (PTMs) including ubiquitination, acetylation and phosphorylation [3]. As an example for the latter, the transcriptionally most potent subunit of NF-κB, p65, is phosphorylated at amino acids 254, 276, 311, 435, 468, 505, 529, 536 by a number of different protein kinases [3]–[6]. PTMs of p65 can regulate interaction with coactivators [7], corepressors [8], promoter-bound degradation [9] and interactions with the basal transcriptional machinery [10]. According to the NF-κB barcode hypothesis that was recently suggested by us the differential modifications of the DNA-binding subunits generate distinct patterns that function to direct transcription in a highly target gene-specific fashion [11].

There is also clear evidence for a pivotal role of NF-κB in chronic inflammation and cancer [12], [13]. In this scenario, NF-κB is activated by cytokines such as IL-1 or TNFα released from cells of the inflammatory tumor microenvironment or by constitutively activated intracellular upstream regulators of IκB kinases (IKKs) within cancer cells. Subsequently, NF-κB induces numerous inflammatory target genes such as chemokines, IL-6 or matrix metallo proteinases which can all be tumor-promoting [14]. However, NF-κB also directly affects cell proliferation by modulating expression of cell cycle-regulatory proteins such as D-type cyclins [15]–[17]. Further, IκBα controls NF-κB transcription factor complex p52/RelB in G1 to S-phase progression [18], and p65 interacts in an inducible manner with cell cycle inhibitor p16INK^4a^ [19]. In contrast, a reciprocal influence of cell cycle signaling pathways on NF-κB-dependent gene expression has been elusive although there is evidence that NF-κB activity and recruitment to proliferative target genes fluctuates during the cell cycle [12].

Cyclin-dependent kinase 6 (CDK6) and its close homologue CDK4 are members of the family of vertebrate cdc-2 related kinases [20], [21]. As they were shown to partner with D-type cyclins and to possess retinoblastoma protein (Rb) kinase activity [22] their main function was considered to relieve Rb-mediated transcriptional repression and to promote G1 to S transition during interphase of the cell cycle [23], [24]. However, genetic evidence has challenged the classical role of CDK4 and CDK6 in G1/S cell cycle transition, as individual or combined deletion of CDK4 and CDK6 remains without impact on cell proliferation [25]–[27]. Moreover, CDK4-activation can occur independent from CDK6 by an unknown upstream proline-directed kinase [28]. This has led to speculations that CDK4 and CDK6 might have yet to discover distinct and unique effector functions that are unrelated to their major substrate Rb and to their role in cell cycle transition [29].

Here we describe the identification of human CDK6 as a NF-κB p65 Ser536 phosphorylating kinase using an unbiased approach. The role of CDK6 for p65 Ser536 phosphorylation was confirmed by gain-of-function and loss-of-function approaches. A transgenic mouse model allowing the lymphocyte-specific activation of CDK6 activity showed increased p65 Ser536 phosphorylation and tumor formation.

## Results

### Identification of CDK6 as a p65 NF-κB Kinase

**Figure 1:**
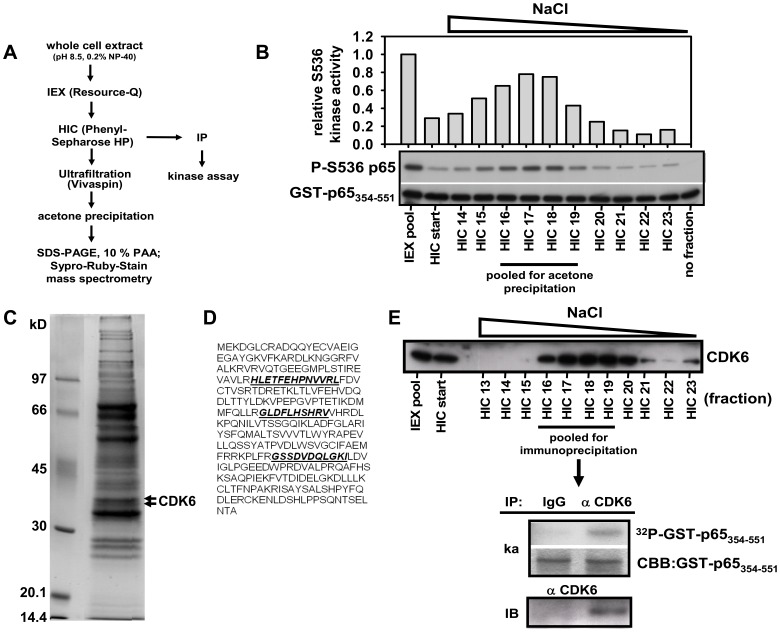
Identification of CDK6 as a NF-κB p65 Ser536 kinase. (**A**) Purification scheme. (**B**) HeLa cells were stimulated with IL-1 for 10 min, lyzed and proteins fractionated by ion exchange chromatography (IEX) on a Resource Q column as previously described [4] followed by hydrophobic interaction chromatography (HIC) on a phenyl sepharose column. Proteins were eluted from phenyl sepharose with a decreasing salt gradient (1 M to 0 M) and individual fractions assayed for *in vitro* phosphorylation of Ser536 using GST-p65_354- 551_ as substrate. Phosphorylated p65 and total p65 protein substrate were detected by immunoblotting. The graph shows quantification of Ser536 kinase activity relative to the starting material (IEX pool). (**C**) Proteins of fractions 16–19 from repeated runs purified up to the HIC step were pooled, concentrated, precipitated and separated in a single lane by SDS PAGE. Proteins were visualized with SyproRuby and 48 segments contained in 45 bands were excised and analyzed by mass spectrometry. Two bands identified as CDK6 are indicated by arrows; the positions of molecular weight marker proteins are indicated. (**D**) Peptide coverage of human CDK6 by three different peptides (underlined) as identified by mass spectrometry. (**E**) p65 Ser536 kinase activity was purified up to the phenyl sepharose step as shown in (B). Upper panel: fractions were analyzed for CDK6 antigen by immunoblotting. Lower panel: fractions 16–19 were pooled and immunoprecipitated with anti CDK6 antibody or control IgG. Immunopurified material was split and analyzed by radioactive *in vitro* kinase assay (ka) using GST-p65_354- 551_ as substrate. Reaction mixtures were separated by SDS-PAGE, stained with Coomassie Brilliant Blue (CBB) and autoradiographed to detect phosphorylation of GST-p65_354- 551_. The other part of the immunoprecipitate was analyzed by immunoblotting (IB) for the presence of CDK6.

We have previously found five distinct protein kinases that phosphorylate the transcriptionally most active NF-κB subunit p65 at Ser536 [4]. They were discriminated based on their elution pattern from ion exchange chromatography of cell extracts of untreated and IL-1-treated cells. All of these kinases were highly specific for Ser536 as they phosphorylated a GST-p65 fusion protein containing amino acids 354-551, but not versions containing Ser536 mutated to alanine [4]. While the p65 Ser536 phosphorylating kinases IKKα, IKKβ, TBK1 and IKKε all eluted at higher NaCl concentrations, an unknown Ser536-specific enzymatic activity eluted from the column very early [4]. Here, we report the purification and identification of this enzyme (Fig. 1A). Extracts from IL-1-treated HeLa cells were chromatographed as shown in Fig. 1A. Peak fractions from Resource Q were pooled and further chromatographed on a phenyl sepharose column. Fractions were assayed using a non-radioactive *in vitro* kinase assay and recombinant GST-p65 (aa 354–551) as substrate (Fig. 1B) exactly as previously described [4]. The fractions containing active p65 Ser536 phosphorylating kinase were pooled, further purified by size exclusion using an ultrafiltration device, concentrated and then separated by SDS-PAGE as shown in (Fig. 1A). This material contained 45 protein bands that stained with SyproRuby (Fig. 1C). Mass spectrometry on tryptic peptides of all proteins present in the sample showed that the fraction contained only one protein kinase, as revealed by the presence of three peptides covering the amino acid sequence of cyclin-dependent kinase 6 (CDK6) (Fig. 1D). Since there are only a few well established substrates for CDK6 such as retinoblastoma protein (Rb) [22], [24], histone H1 [30], Bcl-2 [31], Runx [32] and nucleophosmin [33] we performed additional *in vitro* experiments with the purified fractions to confirm that CDK6 is indeed a direct p65 NF-κB kinase. Antibodies against CDK6 validated the presence of the protein in the phenyl sepharose fractions containing Ser536 kinase activity (Fig. 1B, E). CDK6 immunopurified from these phenyl sepharose fractions displayed p65 phosphorylation as assessed by in *vitro* kinase assays whereas IgG antibodies did not precipitate any p65 NF-κB kinase activity (Fig. 1E).

**Figure 2:**
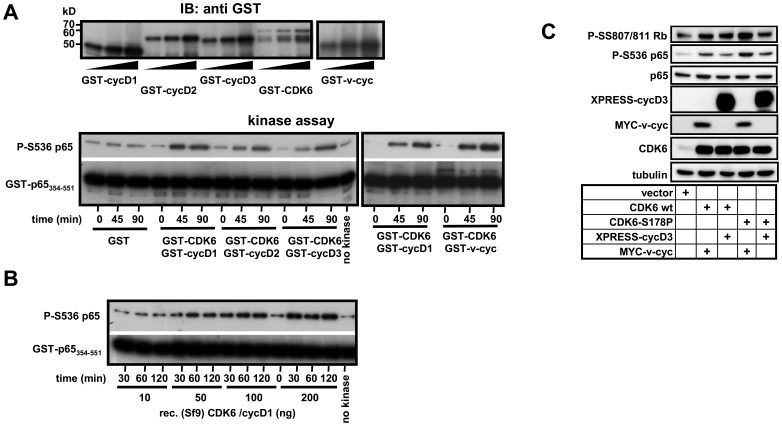
A purified and reconstituted CDK6/cyclin complex phosphorylates NF-κB p65 Ser 536. (**A**) GST fusion proteins of cyclins D1 to D3, viral (v)-cyclin and CDK6 were expressed and purified in *E.coli*. Comparable amounts of GST, GST-cyclins and GST-CDK6 as judged from immunoblotting analysis (upper panel) were mixed with HeLa cell extract to provide CDK-activating kinase CAK as described in [37]. Kinase reactions were performed by addition of ATP. Then, GST alone or activated CDK6/cyclin complexes were purified by addition of GSH-beads, washed and used to phosphorylate the GST-p65_354- 551_ substrate *in vitro*. Phosphorylation of p65 Ser536 was detected by immunoblotting of kinase reaction mixtures (lower panel). Recombinant GST protein or kinase reactions without GST-CDK6/cyclin complexes (labeled no kinase) were used to determine background signals. (**B**) Increasing amounts of a recombinant CDK6/cyclin D1 complex purified to homogeneity from baculovirus-infected insect cells was mixed with GST-p65_354- 551_ and the kinase reaction was allowed to proceed over time. Phosphorylation of p65 at Ser536 was detected by immunoblotting. (**C**) HeLa cells were transfected with the expression vectors for wild type (wt) CDK6, a gain of function mutant (CDK6-S178P) and cyclin D3 or viral (v)- cyclin. 24 h later cells were lysed and phosphorylation of p65 and of Rb and expression of transfected proteins was analyzed by immunoblotting of cell extracts using the indicated antibodies.

CDK6 requires association with cyclins D1-D3 and additional phosphorylations by the cyclin-activating kinase (CAK) complex for full activation [28]. In addition, a viral (v)-cyclin encoded by an open reading frame of Kaposi sarcoma herpesvirus (KSHV) was shown to associate with CDK6 as well as with its close homologue CDK4 [34]. V-cyclin-bound CDK6 has increased activity and is insensitive to inhibition by the CDK inhibitor p16INK^4a^ [30], [35], [36]. To find out if any of the known CDK6/cyclin complexes phosphorylate NF-κB p65 at Ser536, CDK6, cyclins D1–D3 and v-cyclin were expressed in *E. coli* (Fig. 2A, upper panel). Kinase activity of CDK6 in complex with these proteins was assessed using an established *in vitro* coupled kinase assay system by which endogenous CAK activity is provided by addition of cell extracts [37]. After removal of cell extracts, activated GST-CDK6/GST-cyclin complexes were washed to remove contaminating kinases and were used for non-radioactive *in vitro* kinase assays. GST mixed with cell extracts or kinase assays without any addition of GST or GST-CDK6/GST-cyclin complexes were used as negative controls to determine unspecific background signals of the non-radioactive kinase assay. All four activated CDK6/cyclin complexes showed increased *in vitro* phosphorylation of p65 at Ser536 above background signals providing further evidence for CDK6 as a Ser536-specific NF-kB kinase (Fig. 2A, lower panel). Side-by side comparison of kinase activities showed that GST-v-cyclin/GST-CDK6 complexes were slightly more active than GST-cyclinD1/GST-CDK6 complexes (Fig. 2A, lower right panel). To get further independent evidence for CDK6 as a direct Ser536 kinase we used a recombinant CDK6/cyclinD1 complex from baculovirus-infected Sf9 insect cells which according to the commercial supplier contains a nearly homogenously purified CDK6/cyclin D1 complex. This kinase preparation also phosphorylated Ser536 *in vitro* in a time- and dose-dependent manner (Fig. 2B). To further establish CDK6 as a Ser536 kinase in intact cells we ectopically expressed wild type CDK6 or a recently published novel CDK6 mutant which shows increased kinase activity without the requirement of CAK [28] together with cyclin D3 or v-cyclin and assessed the phosphorylation level of endogenous p65 and of the CDK6 substrate Rb. Both, wild type CDK6 and CDK6 S178P increased phosphorylation of p65 NF-κB at Ser536 as well as phosphorylation of Rb (Fig. 2C). The CDK6 mutant CDK6 S178P was slightly more active in phosphorylating p65 Ser536 when compared to the wild type kinase. Furthermore CDK6 S178P kinase activity was highest when it was in conjunction with v-cyclin (Fig. 2C). Collectively, the results shown in Fig. 1 and 2 provide strong evidence for CDK6 as a direct p65 kinase by four independent approaches.

### RNAi-mediated Suppression of CDK6 Affects Cytokine-inducible and Constitutive Phosphorylation of NF-κB p65

**Figure 3:**
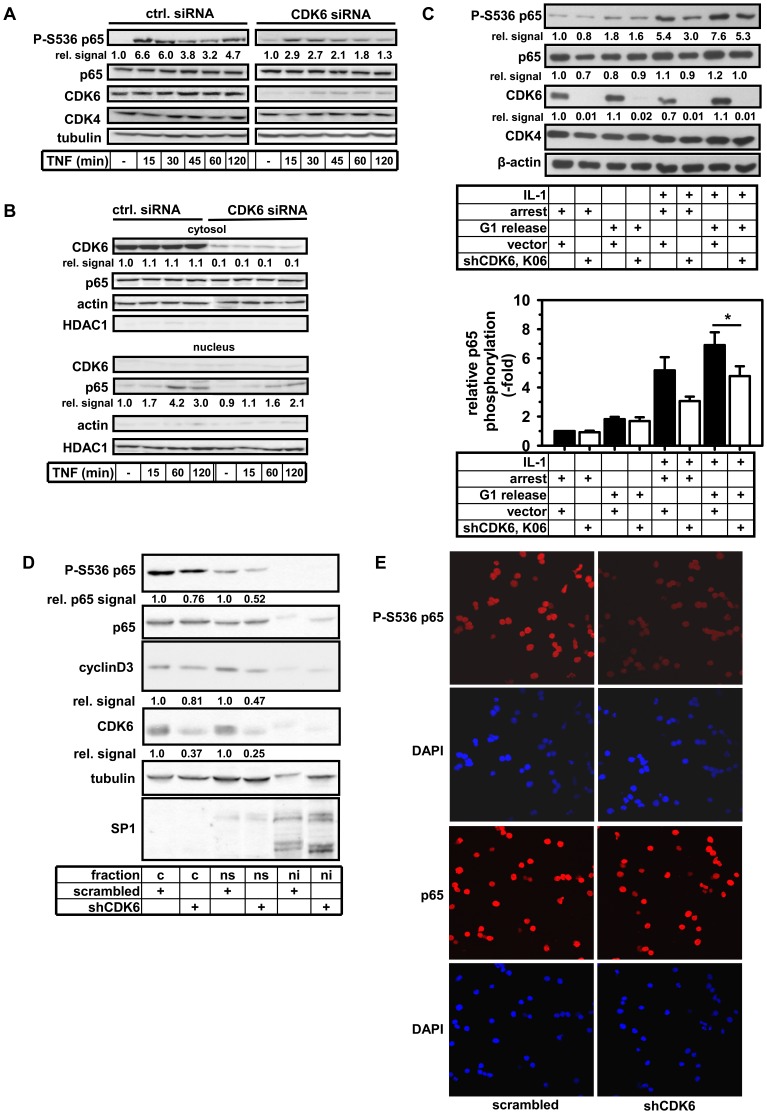
CDK6 contributes to basal and TNF-inducible p65 Ser536 phosphorylation. (**A**) MDCK cells were treated for 24 h with siRNAs directed against CDK6 or adequate scrambled controls followed by treatment with TNFα as indicated. Total cell extracts were analyzed for phosphorylation of p65 and expression of the indicated proteins by immunoblotting. Numbers indicate relative levels of p65 Ser536 phosphorylation. (**B**) The experiment was done as in (A) with the exception that cells were fractionated in nuclear and cytosolic extracts. Extracts were further analyzed for the occurrence of nuclear p65, relative levels are indicated by numbers. Suppression of CDK6 in the cytoplasm was validated by CDK6 antibodies and quantified. Actin and HDAC1 detection served to control the purity of the fractions. (**C**) A Hela cell line stably transfected with shRNAs directed against CDK6 (shCDK6, K06) or cells stably transfected with pSuper-Puro (vector) were subjected to cell cycle arrest by serum deprivation for 48 h (arrest). Thereafter cells were released for 6 h by addition of 20% serum (G1 release). In addition, cells were treated with IL-1 (10 ng/ml) for 30 min as indicated. Total cell extracts were separated by SDS-PAGE in two sets on one gel and were transferred to one membrane. One half of the membrane was probed with antibodies against phospho-Ser536, CDK6, CDK4 and β-actin. The other half was probed with anti p65 antibodies. Numbers indicate relative phosphorylation of p65 or relative protein levels of p65 and CDK6. The graph shows the mean +/− s.e.m. of relative Ser536 phosphorylation normalized to p65 total protein as determined in three independent experiments. The asterisk indicates significant differences (p< = 0.018) as determined by paired t-test. (**D**) Naturally KSHV-infected primary effusion lymphoma (PEL) cells [39] were stably infected with lentiviruses encoding shRNAs directed against CDK6 or scrambled shRNA controls. Cells were fractionated into cytosolic (c), nuclear soluble (ns) and nuclear insoluble (ni) fractions. These extracts were analyzed by immunoblotting with the indicated antibodies. Tubulin was used as a loading control for cytosolic and nuclear soluble fractions and SP1 as a marker for the nuclear insoluble fraction. Numbers indicate relative levels of phospho-Ser536 p65, cyclinD3 and CDK6. (**E**) PEL cells silenced for CDK6 expression as described in (D) were analyzed by indirect immunofluorescence for p65 and p65 Ser536 phosphorylation with specific antibodies, nuclear DNA was revealed by DAPI staining.

We then used RNA-interference to define the functional relevance of CDK6 for TNF-induced p65 phosphorylation. In non-transformed MDCK cells, siRNA-mediated suppression of CDK6 partially reduced cytokine-triggered p65 Ser536 phosphorylation (Fig. 3A). CDK6 knockdown also led to a delayed and reduced nuclear translocation of p65 (Fig. 3B) which is in line with our previous observation that phosphorylation of p65 Ser536 is modulating the nuclear import of p65 [38]. As CDK6 activity is known to be highest in G1 [22] we also tested the requirement of CDK6 for p65 Ser 536 phosphorylation in synchronized tumor cells. In arrested HeLa cells, phospho-Ser536 levels were low but were upregulated by about twofold by serum-mediated cell cycle release (Fig. 3C). IL-1 treatment of cells stimulated p65 Ser536 phosphorylation by five- to six-fold and this effect was slightly enhanced by serum treatment (Fig. 3C). In a stably transfected HeLa cell line with strong CDK6 knockdown, the IL-1-induced increase in Ser536 phosphorylation was inhibited by 30–40% with suppression of the IL-1 effect in G1-released conditions reaching statistical significance (Fig. 3C). Thus the data shown in Fig. 3A to C reveal a contribution of CDK6 to total Ser 536 phosphorylation in intact cells in two cell culture model systems. As CDK6 suppression in these experiments was at least 90%, the remaining Ser536 phosphorylation is most likely due to the activity of other p65 kinases such IKKα/ß. While these experiments show the contribution of CDK6 for cytokine-inducible p65 Ser536 phosphorylation, it was also of interest to investigate whether CDK6 contributes to constitutive p65 phosphorylation as it occurs for example in tumor cells. To address this question we utilized the patient-derived primary effusion lymphoma (PEL) cell line BC-3 [39]. These KSHV-infected B-cell lymphomas critically depend on constitutive NF-κB activity that mediates enhanced production of lymphoma cell survival factors such as IL-6 [40]. These cells also constitutively express v-cyclin that specifically activates CDK6. CDK6 was silenced by expression of a lentivirally delivered specific shRNA and knockdown efficiency was controlled by Western blotting (Fig. 3D). After fractionation of cells into cytosolic (c) and soluble (ns) and insoluble nuclear (ni) fractions, the status of Ser536 phosphorylated p65 was analyzed by immunoblotting. Silencing of CDK6 resulted in a reduced p65 phosphorylation signal in the cells analyzed by immunofluorescence (Figure 3E; right top panel). A modest reduction in p65 phosphorylated at Ser536 and in expression of the NF-κB target gene cyclin D3 was also detected in the cytosolic and the soluble nuclear compartments following CDK6 silencing (Fig. 3D). These results suggest that (i) CDK6 also contributes to permanent p65 Ser536 phosphorylation occurring in tumor cells with constitutive NF-κB activity and (ii) in such cells CDK6 regulates p65 phosphorylation both, in the cytosol and in the nucleus.

### CDK6 Regulates NF-κB-dependent Target Gene Expression

**Figure 4:**
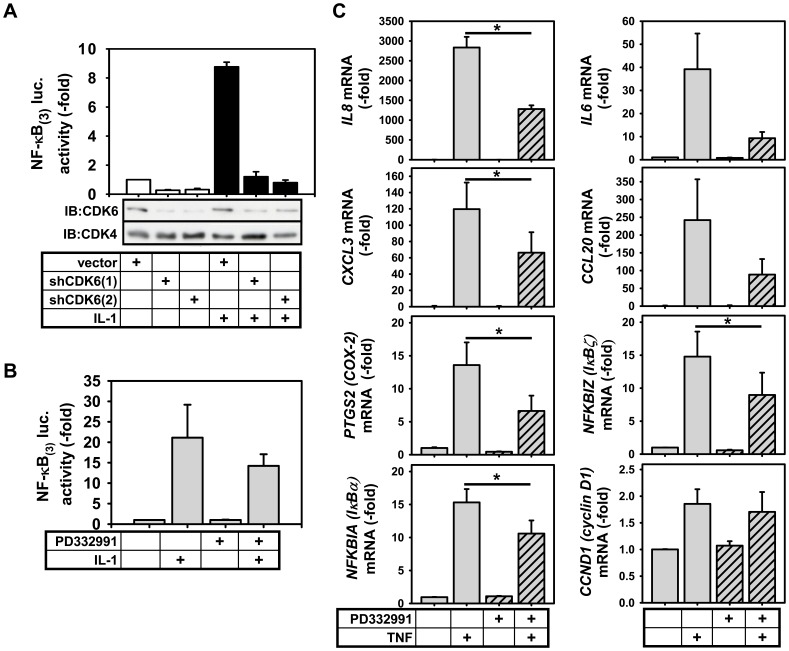
Gene-specific regulation of NF-κB target genes by CDK6. (**A**) HEK293IL-1R cells were transiently transfected with a vector directing the synthesis of two different shRNA duplexes directed at CDK6 or an empty vector control along with a NF-κB-dependent reporter gene and the SV40-ß gal vector to allow for normalization. After 24 h, cells were stimulated for 4 h with IL-1α(10 ng/ml, black bars) or left untreated (white bars). Cells were lysed and luciferase activity was normalized for co-transfected SV40-promoter driven ß-galactosidase. The graph shows the mean luciferase activity +/− s.e.m. from three independent experiments performed in duplicates relative to the vector control. (**B**) HEK293IL-1R cells were transiently transfected with empty vector, NF-κB (3) luc and SV40-ß gal. After 24 h cells were pretretreated with 10 µM PD332991 and then further stimulated for 4 h with IL-1α(10 ng/ml) or left untreated. Shown is the mean luciferase activity +/− s.e.m. from two independent experiments. (**C**) HeLa Fucci cells were arrested for two days and then left untreated or were treated with 10 µM PD332991 (hatched bars) for 30 min followed by 30 min TNFα (20 ng/ml) as indicated. Thereafter, total RNA was prepared and analysed for the expression of the indicated mRNAs by RT-qPCR. Data show the mean –fold change +/− s.e.m. from two independent experiments performed in duplicate. The asterisk indicates significant differences (p< = 0.05) as determined by paired t-test.

To test a possible impact of CDK6 on NF-κB-triggered gene transcription, HEK293IL-1R cells were transiently transfected with vectors encoding two different shRNA duplexes directed at CDK6 or a vector control. Both of them suppressed CDK6 but not CDK4 (Fig. 4A). IL-1-triggered activity of a NF-κB-dependent reporter gene was largely inhibited by the two different shRNAs (Fig. 4A). The importance of protein kinases for signaling can be either due to their enzymatic activity, or alternatively to their function as a scaffold, as exemplified by the Aurora A kinases [41]. As the kinase activity of CDK6 (and of CDK4) can be inhibited by the ATP-competitve inhibitor PD332991 [42] we assessed the effect of this inhibitor on IL-1-induced NF-κB activation and mRNA expression of endogenous genes. PD332991 partially inhibited IL-1-induced NF-κB-dependent reporter gene activation (Fig. 4B). The compound also inhibited TNF-inducible expression of IL-8, CXCL3, CCL20, IL-6, PTGS2 and NFKBIZ expression by about 50% (Fig. 4C), whereas it more weakly affected TNF-induced expression of IκBα and had no impact on inducible cyclin D1 expression (Fig. 4C). These data suggest that the catalytic activity of CDK6 is required for expression of a range of NF-κB dependent inflammatory target genes in a gene specific-manner.

### v-cyclin Activates NF-κB through CDK6 and Induces Tumors in Lymphoid Organs

**Figure 5:**
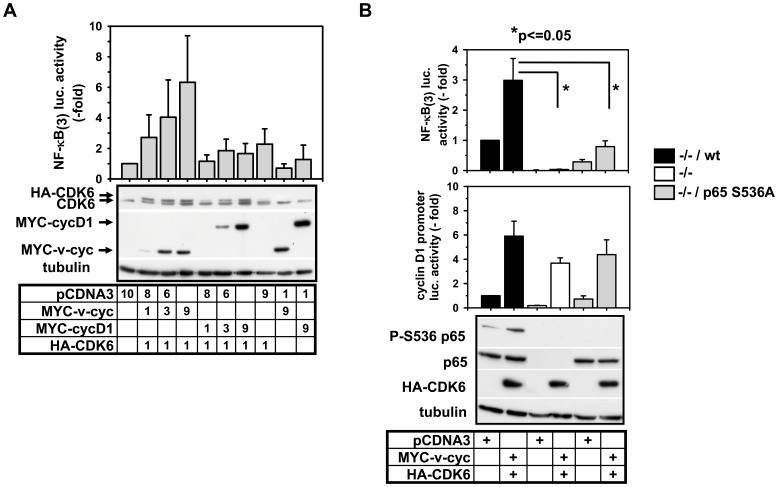
Phosphorylation of Ser536 of p65 by CDK6/cyclin complexes contributes to activation of NF-κB-dependent transcription. (**A**) HEK293IL-1R cells were transiently transfected with the indicated amounts (µg) of expression vectors and a NF-κB-dependent luciferase gene. After 24 h cells were lyzed and luciferase activity was normalized for co-transfected SV40-promoter driven ß-galactosidase. The graph shows the mean luciferase activity +/− s.e.m. from three independent experiments performed in duplicates relative to the vector control. Expression of cotransfected MYC-cyclin D1, v-cyclin derived from Kaposi’s sarcoma herpesvirus (MYC-v-cyclin) and HA-CDK6 was analyzed by immunoblotting using anti MYC or anti CDK6 antibodies for all experiments in parallel. One representative blot is shown. (**B**) NF-κB p65-deficient MEFs stably reconstituted with wild type p65 (−/−/wt) or the Ser536A mutant (−/−/Ser536A) as described [64] were transiently transfected with empty vector or HA-CDK6 plus v-cyclin expression vectors. Cotransfected luciferase reporter genes were either controlled by three NF-κB binding sites (upper graph) or alternatively by the cyclin D1 promoter (lower graph). After 24 h cells were lyzed, luciferase activity was determined and normalized for co-transfected SV40-promoter driven ß-galactosidase activity. Shown is the mean luc. activity +/− s.e.m. from three (lower graph) or four (upper graph) independent experiments performed in duplicates relative to the vector control. The lower panel shows a representative immunoblot for the detection of p65 wt and Ser536Ala mutant, p65 Ser536 phosphorylation and ectopically expressed CDK6. Asterisks indicate significant changes of reporter gene activity.

We then investigated a functional role of CDK6 in NF-κB activation by a gain-of-function approach resembling pathophysiological conditions where CDK6 is overexpressed or constitutively activated [26]. Towards this goal we coexpressed v-cyclin or cyclin D1 together with CDK6 and measured the impact on NF-κB reporter gene activity. Ectopically expressed CDK6 alone caused a twofold increase in NF-κB activation (Fig. 5A). However, coexpression of increasing amounts of v-cyclin allowed a dose-dependent increase of NF-κB-dependent transcription (Fig. 5A). To investigate whether NF-κB p65 phosphorylation contributes to the stimulatory effect of CDK6 on NF-κB activity p65-deficient murine embryonic fibroblasts (MEFs) were transfected to express wild type p65 or the non-phosphorylatable p65 S536A mutant along with v-cyclin/CDK6 and two different reporter genes. The NF-κB-dependent reporter gene was stimulated by threefold upon expression of v-cyclin and CDK6 (Fig. 5B upper), conditions that also trigger p65 Ser536 phosphorylation. No activation was seen in the absence of p65, and the cells reconstituted with the p65 Ser536A mutant showed impaired basal and v-cyclin/CDK6-induced NF-κB activation. In contrast, a reporter gene under the control of the cyclin D1 promoter containing a functional NF-κB binding site [16], [17,17] showed a different behavior, as p65 Ser536 phosphorylation was of minor relevance for CDK6/v-cyclin-triggered gene induction (Fig. 5B lower).

**Figure 6:**
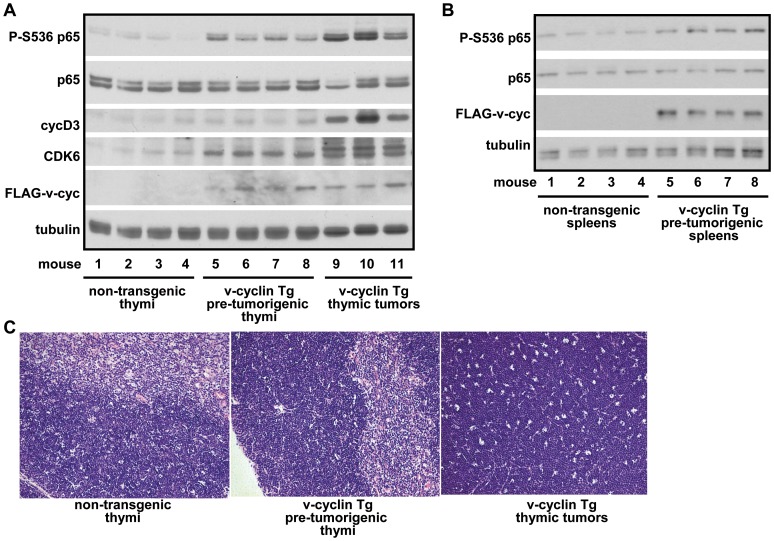
p65 Ser536 phosphorylation is increased in v-cyclin expressing B-cell lymphomas, pre-tumorigenic lymphoid organs and further enhanced in thymic tumors. (**A**, **B**) Whole cell extracts were prepared from thymic (A) and splenic (B) lymphocytes isolated from non-transgenic littermates and mice expressing FLAG-v-cyclin under the control of the Eμ promoter which targets v-cyclin expression to B- and T-cell compartments [44], [45]. Lymphocytes from control thymi and spleens, pre-tumorigenic thymi and spleens, as well as thymic tumors were analysed by immunoblotting with antibodies against p65 phosphorylated at Ser536 (P-S536 p65), p65, CDK6, anti-FLAG (for v-cyclin) and cyclin D3. Anti-tubulin was used as loading control. Numbers indicate individual mice. (**C**) H&E staining of tissue sections corresponding to thymic tissues as described above.

To address the role of CDK6/v-cyclin complex in p65 Ser536 phosphorylation and NF-κB activation under pathophysiological conditions, we analyzed Eμ-v-cyclin transgenic mice. In this model, v-cyclin expression is targeted to B- and T-lymphocyte compartments by a tissue specific promoter leading to development of splenic and thymic tumors in about 10–20% of these mice [43]–[45]. Indeed, increased levels of phosphorylated p65 Ser536 were detected in lymphocytes from pre-tumorigenic thymi and thymic tumors (Fig. 6A) as well as in pre-tumorigenic spleens (Fig. 6B) of the v-cyclin transgenic animals as compared to the thymic or splenic lymphocytes isolated from non-transgenic littermates. Importantly, the v-cyclin expressing transgenic mice demonstrating increased p65 Ser536 phosphorylation (Fig. 6A,B) also developed lymphoid tumors (Fig. 6C). Increased p65 Ser536 phosphorylation in the tumors correlated with increased levels of CDK6 and the NF-κB target gene cyclin D3 (Fig. 6A). These data strengthen the role of CDK6 and its viral cyclin partner in the phosphorylation of p65, and suggest the occurrence of increased NF-κB activity in the affected organs of the Eμ-v-cyclin transgenic mouse.

## Discussion

NF-κB p65 Ser535 phosphorylation is executed by a surprisingly large number of different kinases and phosphatases [46] raising the possibility that the various kinases have partially redundant functions and are of special relevance in specific signaling pathways. It is presently not clear whether CDK6 phosphorylates p65 in the cytosol or in the nucleus, as both proteins occur in these two compartments. Whereas Ser536 phosphorylation of cytosolic p65 will increase the efficiency of nuclear import (see Fig. 3B and [38]) this phosphorylation will serve other purposes in the nucleus. NF-κB p65 Ser536 phosphorylation regulates interaction with corepressors and coactivators such as TAFII31, one of the subunits of TFIID thus directly contributing to increased transactivation of genes like IL-8 [4]. It is generally observed that posttranslational modifications of the NF-κB DNA-binding subunits have no global effects on transcription but rather serve to control gene expression in a highly target gene specific manner [11], [47]. This raises the possibility that NF-κB kinases can also modify further substrates (chromatin components, coregulatory proteins) relevant for the expression of inflammatory genes. It will therefore be interesting to study in the future whether the role of CDK6 for a subset of NF-κB-dependent target genes relies entirely on its ability to phosphorylate Ser536 or whether also other mechanisms are involved. Another implication of this study stems from the natural fluctuation of CDK6 kinase activity which is maximal at the G1/S transition. This raises the possibility that the expression of CDK6-dependent proinflammatory genes is differentially regulated in various cell cycle phases.

Chronic inflammation and cancer are intimately linked underscoring the importance to unravel the underlying mechanisms of molecular and physiological cross-talk [13], [14]. The CDK6 gene is frequently amplified or overexpressed in a variety of human tumors [29], [48] such as glioblastoma [49] and human lymphoid malignancies [48], [50], [51]. CDK6 or its close counterpart CDK4 also act as oncogenes in tumors expressing mutant versions or devoid of cell cycle inhibitors such as p16INK^4a^ [52]. In most cases, the contribution of CDK6 to tumor phenotypes has been assessed in conjunction with CDK4, particularly in studies using PD332991 which inhibits both kinases [42]. While there is ample evidence that the transforming properties of CDK4/6 are transmitted through phosphorylation of Rb [26], CDK4/6 can also contribute to tumorigenesis in the absence of Rb [53] by phosphorylating as yet unidentified substrates [54]. In line with this, a large number of additional substrates containing at least two CDK consensus sites were recently identified *in vitro* [55]. Ser536 of p65 NF-κB is not part of the classical CDK consensus site Ser/Thr-Pro-X-Lys/Arg. A recent large scale *in silico* analysis concluded that even the minimal CDK motif Ser/Thr-Pro is equally often found in CDK6 substrates as well as in non-substrates underscoring the difficulty in predicting CDK6 phosphorylation sites [56]. These authors also found that CDKs are more likely to phosphorylate Ser/Thr residues in flexible, unstructured regions of proteins [56]. The p65 transactivation domain containing Ser536 adopts such an unstructured conformation [57]. Hence, our data suggest that Ser536 of p65 represents a novel, unusual CDK6 phosphorylation site. Such a phenomenon has been found for other proline-directed kinases, e.g. Ser423 in the motif Ala-His-Ser-Ala of TAB1 is phosphorylated by p38 MAPK [58]. In addition to having substrates other than Rb, there is also increasing evidence for functional differences between CDK4 and CDK6 [28], [50], [54], [59], [60]. Thus, CDK6 may affect uncontrolled proliferation during tumor development by Rb-independent mechanisms and accordingly our study unraveled a novel connection between CDK6 and NF-κB. Depending on the underlying genomic alterations, p65 transactivation activity may be increased by a variety of oncogenic kinases such as IKKε [61] or CDK6 as shown in this study. This predicts that increased abundance or activity of CDK6 will amplify both, inflammation and tumor progression.

## Materials and Methods

### Cell Lines

HeLa cells were from ATCC and HEK293 cells stably expressing the IL-1 receptor (HEK293IL-1R) have been described [4], [62]. HeLa Fucci cells were from A. Miyawaki [63]. NF-κB p65-deficient murine embryonic fibroblasts (MEFs) and cells stably reconstituted with p65 or p65 (Ser536A) were from H. Nakano [64]. Cells were cultured in Dulbecco’s modified Eagle’s medium, complemented with 10% fetal calf serum, 2 mM L-glutamine, 100 U/ml penicillin, 100 µg/ml streptomycin. The BC-3 PEL cell line [33], [65] was kindly provided by E. Cesarman (Cornell Medical College, NY) and cultured as described previously [44]. MDCK cells (European Collection of Cell Cultures) were cultured in RPMI 1640 medium (PAA Laboratories) supplemented with 10% fetal calf serum and penicillin/streptomycin. All cell lines were kept in a humid atmosphere at 37°C with 5% CO_2_.

### Materials

Recombinant human TNFα was from R&D Systems or Hoelzel. IL-1α was made as described [4]. PD332991 was a gift from Pfizer or was purchased from Selleck Chemicals. Recombinant CDK6/cycD1 complex (Product No.: 0051-0143-1, ProQinase) was made in Sf9 cells and purified to near homogeneity as judged by Coomassie staining and western blotting by the manufacturer. Antibodies against the following proteins/peptides were used: actin (JLA20, Calbiochem) or (Santa Cruz (sc)-1616), CDK4 (C22, sc-260), CDK6 (C21, sc-177, DCS-90, sc-56282), GST (Z5, sc459), cyclin D1 (M20, sc-718), SP-1 (PEP2, sc-59), cyclin D3 (2936, Cell Signaling (c.s.)), P-Ser536 p65 (3031, 3033 (93H1), 3036 (7F1), c.s.), p65 (C-20, sc-109, sc-372, F-6, sc-8008), mouse IgG (sc-2025), rabbit IgG (sc-2027), P-SS807/811 Rb (9308, c.s.), tubulin (TU-02, sc-8035) or T4026 or GTU-88 (Sigma), HA, (clone 12CA5, Roche), MYC (9E10, Roche), FLAG M2 (F1804, Sigma), HDAC1 (5356, c.s.).

Horseradish peroxidase-coupled secondary antibodies: goat anti-mouse IgG and goat anti-rabbit IgG were from Dako Cytomation or Dianova, rabbit anti chicken IgG (A-9046) was from Sigma, IgG TrueBlot was from eBioscience. GST-fusion proteins were expressed in E.coli BL21 and purified by standard procedures.

### Plasmids, Transfections, Stable Cell Lines and Reporter Gene Assays

pCDNA3-CDK6-wt, pCDNA3-CDK6-S178P, pCDNA3-HIS-Xpress-Cyclin D3 were from Pierre Roger [28], pGEX-based expression vectors for GST-cyclin D1, D2 and D3 were from Yue Xiong [37], pGEX-CDK6, pGEX-v(KSHV)-cyclin, pCDNA3-MYC-v(KSHV)-cyclin, pCDNA3-MYC-cyclin D1, pCI-Neo-HA-CDK6 were from Philip Kaldis [36]. The human cyclin D1 promoter(-1745)-pA3.Luc was from Richard G.Pestell [16]. For generation of pSuper-Puro-shCDHK6 plasmids (Fig. 3C and Fig. 4A), the following oligonucleotides were cloned into Bgl II/Hind III sites of the vector:

shCDK6(1): forward primer, 5′-GATCCCC**AGTAGTGCATCGCGATCTA**ttcaagaga**TAGATCGCGATGCACTACT**TTTTTGGAAA-3′, reverse primer, 5′-AGCTTTTCCAAAAA**AGTAGTGCATCGCGATCTA**tctcttgaa**TAGATCGCGATGCACTACT**GGG. shCDK6(2): forward primer, 5′-GATCCCC**GCAGAAATGTTTCGTAGAA**ttcaagaga**TTCTACGAAACATTTCTGC**TTTTTGGAAA, reverse primer, 5′- AGCTTTTCCAAAAA**GCAGAAATGTTTCGTAGAA**tctcttgaa**TTCTACGAAACATTTCTGC**GGG).

Sequences complementary to human CDK6 are shown in bold. For generation of stable HeLa cell lines (Fig. 3C), pSuper-Puro-shCDK6(2) or empty pSuper-Puro plasmids were transiently transfected by the calcium phosphate method and pools of cells selected and maintained in 1 µg/ml puromycin. The monoclonal cell line K06 was obtained by limiting dilution.

Transient transfection of siRNA (sequences available on request) against CDK6 was carried out in OptiMEM serum-free medium (Invitrogen) using 50 ng siRNAduplexes and siLentFect reagent (Biorad). After 6 h the medium was removed and cells were cultured in serum-containing medium. Subsequently, 24 h after transient transfection cells were washed twice with PBS and stimulated with TNFα as indicated. GST-p65_354-355_ and NF-κB_(3)_-Luc., have been described [4]. HEK293IL-1R or HeLa cells were transiently transfected by the calcium phosphate method. p65−/− cells were transfected using Rotifect (Roth) as described [4]. Equal amounts of plasmid DNA within each experiment were obtained by adding empty vector. For determination of promoter activity, cells were seeded in 6-well plates and were transfected with 0.25 µg of luciferase reporter plasmids and 0.5 to 1 µg of pSV-ß-gal. ß-Galactosidase activity was determined using reagents from Clontech to allow normalization of luciferase activity in different transfections.

### Cell Lysis and Immunoblotting

Whole cell extracts were prepared upon lysis of cells in Triton cell lysis buffer (10 mM Tris, pH 7.05, 30 mM NaPP_i_, 50 mM NaCl, 1% Triton X-100, 2 mM Na_3_VO_4_, 50 mM NaF, 20 mM ß-glycerophosphate and freshly added 0.5 mM PMSF, 0.5 µg/ml Leupeptin, 0.5 µg/ml pepstatin, 1 µg/ml microcystin) as previously described [66]. Cell lysates were subjected to SDS-PAGE on 6–9% gels and immunoblotting was performed as described below. MDCK cells were washed twice with ice-cold PBS and lysed in RIPA-buffer as described previously [67]. To prepare cytosolic and nuclear fractions [68], cells were trypsinized, washed twice with ice-cold PBS and harvested by centrifugation (500×g, 5 min, 4°C). Then cell pellets were resuspended in hypotonic buffer. After centrifugation (2,300×g, 1 min, 4°C) supernatants were used as cytoplasmic fractions. Nuclear pellets were washed twice with hypotonic buffer and lysed in hypertonic buffer with brief vortexing. Preparation of soluble nuclear extracts was carried out by centrifugation (16,100×g, 5 min, 4°C).

Immunoblotting was performed essentially as described [4]. Proteins were separated on 6–10% SDS-PAGE and electrophoretically transferred to PVDF membranes (Millipore). After blocking with 2% or 5% dried milk in Tris-HCl-buffered saline/0.05% Tween (TBST) for 1 h, membranes were incubated for 2–24 h with primary antibodies, washed in TBST and incubated for 1–2 h with horseradish peroxidase-coupled secondary antibodies. Proteins were detected by using enhanced chemiluminescence (ECL) systems from Perbio, Pierce, Millipore or GE Healthcare on x-ray films or the VersaDoc Imaging System (Biorad).

### Purification of the p65 Ser536-phosphorylating Kinase

Three to four T175-cm2 flasks of HeLa cells were stimulated by IL-1 (10 ng/ml) for 10 min at 37°C/5% CO_2_. Thereafter, cells were harvested by scraping, followed by centrifugation (5 min, 500×g, 4°C), washed twice in ice-cold phosphate-buffered saline containing 10 mM NaF and resuspended in 20 mM Tris, pH 8.5, 20 mM beta-glycerophosphate, 20 mM NaF, 0.1 mM Na_3_VO_4_, 0.5 mM EGTA, 0.5 mM EDTA, 0.1% (w/v) Nonidet P-40, 2 mM Dithiothreitol, 10 µM E64, 2.5 µg/ml leupeptin, 1 mM phenylmethylsulfonyl fluoride, 1 µM pepstatin, and 400 nM okadaic acid. Cells were broken mechanically by repeated rigorous vortexing and intermediate incubation on ice for 30 min. Then the lysate was cleared at 10,000×g (13,000 rpm) for 15 min at 4°C. Supernatants were frozen in liquid nitrogen and stored at −80°C. Lysate containing approximately 4–6 mg total protein was diluted into 2.5 ml of buffer A (20 mM Tris, pH 8.5, 20 mM ß-glycerophosphate, 20 mM NaF, 0.1 mM Na_3_VO_4_, 0.5 mM EGTA, 0.5 mM EDTA, 0.05% Nonidet P-40, 2 mM dithiothreitol), filtered through an 0.2 µM membrane (Sartorius) and loaded onto a 1 ml Resource Q column (GE Healthcare) equilibrated in buffer A. After washing the column, proteins were eluted with a linear salt gradient (0–0.75 M NaCl in 1 6 ml). 1 ml fractions were collected, frozen (N_2_) and stored at −80°C until further use.

The kinase of interest eluted with an activity maximum at about 0.1 M NaCl. Active Fractions (fractions F5–F8) of four to six ResourceQ-runs were pooled and prepared for hydrophobic interaction chromatography conditions by slowly adding one fourth of sample volume of starting buffer (0.48 M Tris/HCl, pH 6.7, 4 M NaCl, 20 mM ß-glycerophosphate, 10 mM NaF, 4 mM DTT), resulting in a final sample pH of 7.3 and an initial NaCl concentration at 1 M. The sample was filtered and applied onto a 1 ml Phenyl-Sepharose HP column (GE Healthcare) equilibrated with running buffer C (20 mM Tris/HCl, pH 7.3, 1 M NaCl, 20 mM mM ß-glycerophosphate, 20 mM NaF, 0.25 mM EGTA, 0.25 mM EDTA, 2 mM DTT). Elution of the kinase was achieved by a decreasing NaCl- gradient (1 M to 0 M NaCl in 16 ml) using Buffer D (Buffer C without NaCl). Peak activity was observed at a NaCl concentration of approximately 0.4 M NaCl. Active fractions (H16–H19) of 10 lysates were pooled and concentrated by ultrafiltration (vivaspin 2 (10,000 MWCO) RC Sartorius Stedim) by the factor 12 related to the initial volume. To prevent protein unspecific loss of protein the membrane of the ultrafiltration device was coated with 5% (w/v) Triton X-100 according to manufacturers instructions. For sample preparation prior to preparative SDS-PAGE further protein concentration was performed by acetone precipitation (by adding 4 volume parts acetone per one volume sample, followed by incubation at −20°C for 4 h and a subsequent centrifugation (13,000 rpm, 4°C, 20 min)). For SDS-PAGE (Gel: 10% PAA) resulting pellets were resuspended in reducing 1xSDS-PAGE-sample buffer, boiled for 23 min. and loaded onto the gel (loaded amount was the equivalent of five lysates). Sypro Ruby stain of the gel was performed overnight according to manufactureŕs protocol. Detected protein bands were excised and detected by mass spectrometry.

### *In vitro* Kinase Assays

For the kinase assays, 10 µl of HIC fractions or 10 µl of recombinant CDK6/cycD1 were mixed with 1 µg of GST-p65_354-551_ in 10 µl T and 10 µl of kinase buffer (150 mM Tris, pH 7.4, 30 mM MgCl_2_, 600 µM ATP). After 30 min at 30°C reaction mixtures were separated by SDS-PAGE and phosphorylation of p65 was detected by immunoblotting with the anti P-Ser536 antibodies. Substrate loading was controlled by re-probing with anti p65 antibodies.

For IP of HIC-purified material, 120 µl of pooled HIC fractions 16–19 were diluted to 500 µl with Resource Q buffer A, pH 7.3, including 1% BSA, 0.05% NP-40, 0.2 mM DTT and 1 µg of rabbit IgG or anti CDK6 antibody was added for 2 h at 4°C. Thereafter samples were precipitated with 40 µl of pre-equilibrated protein G sepharose and washed 2x in 500 µl of IP buffer including 0.2% NP-40, 1 mM DTT. Then, 1/5 of the sample was removed for immunoblotting. Beads were spun down and resuspended in 10 µl of IP buffer. 20 µl of kinase buffer including 1.5 µg of GST-p65_354-551_, 4 µCi ^32^P-ATP and 15 µM ATP. After 45 min at 30°C samples were separated by SDS-PAGE and phosphorylated GST-p65 was detected by autoradiography.

For assessing the *in vitro* activites of CDK6/cyclin complexes, HeLa cells were resuspended in cyclin-activating kinase (CAK) buffer (80 mM Hepes, pH 7.4, 15 mM MgCl_2_, 1 mM Na_3_VO_4_, 10 mM NaF, 10 mM ß-glycerophosphate, 2 mM DTT, 1 mM PMSF, Roche protease inhibitors). Cells were broken mechanically by repeated rigorous vortexing and supernatants recovered by centrifugation. 50 µl of lysate (250 µg) were mixed with 2 µg of GST-CDK6 plus 2 µg of GST-cyclins in CAK buffer including 1 mM ATP and 0.1% NP-40 for 23 h at 30°C. GST-fusion proteins were purified by addition of GSH sepharose for 30 min at 4°C, washed 3x and eluted in 100 µl of CAK buffer plus 10 mM GSH for 15 min at 30°C. 10 µl of the eluate was mixed with 1 µg of GST-p65_354-551_ in 20 µl kinase reaction buffer including 200 µM ATP. After incubation at 30°C for the indicated times, proteins were separated by SDS-PAGE and phosphorylation of GST-p65 was detected by immunoblotting.

Further details on *in vitro* kinase assays for phosphorylation of GST-p65_354-551_ are described in [4] and in [37].

### Protein Identification by Tandem Mass Spectrometry

Sypro ruby-stained gel bands were excised with a scalpel, and proteins were digested in gel with trypsin, using an Investigator Progest robot (Genomic Solutions, Huntingdon, UK). Samples were analyzed by high performance liquid chromatography coupled to electrospray ionisation tandem mass spectrometry (HPLC ESI MS/MS). HPLC was carried out on a CapLC liquid chromatography system (Waters, ManchesterUK). Aliquots (6 µL) of peptide mixtures were injected onto a Pepmap C18 column (300 µm×0.5 cm; LC Packings, Amsterdam, The Netherlands) and eluted with an acetonitrile/0.1% formic acid gradient to the nanoelectrospray source of a Q-Tof spectrometer (Micromass, Manchester, UK) at a flow rate of 1 μ/min. The spray voltage was set to 3500V and data dependent MS/MS acquisitions were performed on precursor peptides with charge states 2, 3, or 4 over a survey mass range 440–1400 using argon collision gas. The recorded product ion spectra were transformed into a singly charged *m/z* axis using a maximum entropy method (MaxEnt3, Waters, UK), and centroided peaklist (pkl) files were extracted using the MassLynx component peptide auto (Waters, Manchester UK). Proteins were identified by correlation of uninterpreted spectra to entries in SwissProt (Release 2010_04: 516,081 entries) using a local installation of Mascot (version 2.2: www.matrixscience.com). MS/MS ion searches specified up to two missed cleavages per peptide, a precursor mass tolerance of ±0.5 Da and a fragment ion mass tolerance of ±0.5 Da. Carbamidomethylation of cysteines and methionine oxidation were specified as fixed and variable modifications respectively. Criteria for protein identification: MS/MS based peptide and protein identifications were validated using Scaffold (Proteome Software Inc., Portland, Oregon: version 3.01). Peptide identifications were accepted if they could be established at greater than 95.0% probability as specified by the Peptide Prophet algorithm [69]. Protein Identifications were accepted if established at greater than 99.0% probability and contained at least 2 matched peptides. Protein probabilities were assigned by the Protein Prophet algorithm [70].

### RT-qPCR

1 µg of total RNA was prepared by column purification (Qiagen) and transcribed into cDNA using Moloney murine leukemia virus reverse transcriptase (MBI) in a total volume of 20 µl. 1–2 µl/well of this reaction mixture was used to amplify cDNAs using assays on demand (ABI) and an ABI7500 Fast real time PCR instrument. The threshold value ct for each individual PCR product was calculated by the instrument’s software, and ct values were normalized by subtracting the ct values obtained for ß-actin. The resulting Δct values were then used to calculate relative changes of mRNA expression as ratio (R) of mRNA expression of stimulated/unstimulated cells according to the equation: R = 2^−(Δct(stimulated)−Δct(unstimulated))^.

### Lentiviral Production and Transduction

Production of lentiviral supernatants and CDK6 silencing in BC-3 cells were performed as described earlier [45].

### Subcellular Fractionation and Immunoblotting

Subcellular fractionation of BC-3 cells was performed as described earlier [71].

### Eμ-v-cyclin Transgenic Mice and Analysis of Mouse Lymphocytes

Eμ-v-cyclin transgenic mice were kindly provided by Emmy Verschuren and Gerard Evan (Cancer Research Institute, University of California) and were maintained as described earlier [44], [45]. Spleens from 5.5-week old pre-tumorigenic mice were obtained by dissection. Tissue was disaggregated by pressing through a 70 µm nylon mesh cell strainer (BD Falcon) in RPMI containing 10% FCS to obtain a single cell suspension. Splenic erythrocytes were eliminated by incubation for 5 min at room temperature in ACK buffer (155 mM NH_4_Cl, 10 mM KHCO_3_, and 0.1 mM EDTA, pH 7.8). Lymphocytes were pelleted and washed once with PBS before lysis. Mouse lymphocytes were lysed to Urea-Tris Buffer (UTB) (9 M Urea, 75 mM Tris-HCl, pH 7.5, 0.15 M 2-mercaptoethanol, complete proteinase inhibitor cocktail, phosphatase inhibitory cocktail (PhosphoSTOP, Roche)), homogenized by sonication and cleared by centrifugation. Proteins (50 µg of spleens and PEL cells, 75 µg of thymi) were fractionated by SDS-PAGE and were transferred to nitrocellulose membranes. Membranes were probed with antibodies as described above.

### Statistics

Statistics were calculated by the Mann-Whitney Rank test or paired t-test using Sigma Plot, version 11.0. Bands detected by immunoblotting were quantified using ImageJ (http://rsbweb.nih.gov/ij/).
